# Isolation and Characterization of Potential *Salmonella* Phages Targeting Multidrug-Resistant and Major Serovars of *Salmonella* Derived From Broiler Production Chain in Thailand

**DOI:** 10.3389/fmicb.2021.662461

**Published:** 2021-05-28

**Authors:** Wattana Pelyuntha, Ruttayaporn Ngasaman, Mingkwan Yingkajorn, Kridda Chukiatsiri, Soottawat Benjakul, Kitiya Vongkamjan

**Affiliations:** ^1^Faculty of Agro-Industry, Prince of Songkla University, Songkhla, Thailand; ^2^Faculty of Veterinary Science, Prince of Songkla University, Songkhla, Thailand; ^3^Division of Pathology, Faculty of Medicine, Prince of Songkla University, Songkhla, Thailand; ^4^Faculty of Animal Science and Technology, Maejo University, Chiang Mai, Thailand; ^5^International Center of Excellence in Seafood Science and Innovation, Faculty of Agro-Industry, Prince of Songkla University, Songkhla, Thailand; ^6^Department of Biotechnology, Faculty of Agro-Industry, Kasetsart University, Bangkok, Thailand

**Keywords:** animal farm, antibiotic resistance, biocontrol, broiler, phage cocktail, *Salmonella*, phage lysis, phage therapy

## Abstract

*Salmonella* is a major foodborne pathogen that causes foodborne disease in humans through consumption of contaminated foods, especially those of animal origin. Multiple *Salmonella* strains are antibiotic-resistant due to the common use of antibiotics in farm animals, including broiler farms. In this study, an alternative strategy using phage-based treatment was evaluated against *Salmonella* isolated from the broiler production. The prevalence of *Salmonella* spp. showed up to 46.2 and 44.4% in bedding samples from the broiler farms located in eastern and southern Thailand, respectively. Overall, 21 samples (36.2%) were positive for *Salmonella* and eight serovars were recovered from cloacal swabs, bedding materials (rice husk), and boot swabs collected from five farms. Up to 20 *Salmonella* phages were isolated from seven water samples from wastewater treatment ponds, a river, and a natural reservoir in Songkhla province. Isolated phages were investigated, as well as their lysis ability on eight target *Salmonella* serovars derived from broiler farms, five foodborne outbreak-related serovars, and 10 multidrug-resistant (MDR) serovars. All phages showed a strong lytic ability against five serovars of *Salmonella* derived from broiler farms including Kentucky, Saintpaul, Schwarzengrund, Corvalis, and Typhimurium; three foodborne outbreak serovars including Enteritidis, Typhimurium, and Virchow; and eight MDR serovars including Agona, Albany, Give, Kentucky, Typhimurium, Schwarzengrund, Singapore, and Weltevreden. Three phages with the highest lysis potential including vB_SenS_WP109, vB_SenS_WP110, and vB_SenP_WP128 were selected for a phage cocktail preparation. Overall, a phage cocktail could reduce *Salmonella* counts by 2.2–2.8 log units at 6 h of treatment. Moreover, *Salmonella* did not develop a resistant pattern after being treated with a phage cocktail. Findings here suggest that a phage cocktail is an effective biocontrol to combat *Salmonella* derived from broiler production chain, other serovars linked to foodborne outbreaks, and MDR serovars.

## Introduction

Salmonellosis is one of the most common foodborne infections worldwide caused by consumption of contaminated foods with *Salmonella* spp. and becoming an important public health concern (Eng et al., 2015). Poultry meat and its products have served as major sources of *Salmonella* spp., which have caused human and animal diseases as well as economic losses to the poultry industry (Barbour et al., 2015). In Thailand, *Salmonella* contamination in broilers and poultry meat has been previously reported (Sripaurya et al., 2019). Although *Salmonella* contamination in broiler farms (at the beginning of the poultry production chain) is commonly reported, Dookeran et al. (2012) observed that broiler carcass contamination with *Salmonella* spp. increased during transportation from the farm to the processing plant, during processing, and at retail outlets.

For the poultry industry, controlling the presence of *Salmonella* is crucial for food safety as well as for preventing the spread of antimicrobial resistance (Pulido-Landínez, 2019). Antimicrobial agents are widely used for growth promotion or treatment purposes (World Health Organization (WHO), 2018), which can lead to the occurrence of antimicrobial-resistant strains in broilers and broiler meat. These will link to a potential source for transmission to human since antibiotic-resistant *Salmonella* isolated at the farm level may spread to humans through direct contact or contaminated meat (Dolejska et al., 2013). Overall, antimicrobial-resistant *Salmonella* strains are frequently encountered worldwide and the proportion of antimicrobial-resistant dramatically increased over the past decade (World Health Organization (WHO), 2018). To decrease the risk factors that are related to *Salmonella* contamination in poultry meat production, the steps of *Salmonella* elimination from poultry cultivation to processing and handling (from farm to table) are necessary (Vandeplas et al., 2010; Alum et al., 2016).

Bacteriophages (phages) are viruses of bacteria and not harmful to humans and animals. Phages have been used to treat bacterial infection caused by MDR microorganisms and treat against several foodborne pathogens (Goodridge and Bisha, 2011; Dy et al., 2018) with a strong bactericidal effect and high specificity. Phage has become an attractive approach to combat *Salmonella* in broilers or broiler meat due to its high specificity and effortless in administration or application (Lim et al., 2012; Nabil et al., 2018). In addition, several previous studies have confirmed that a phage cocktail composed of several phages was more effective in controlling *Salmonella* spp. than one phage alone (Goodridge, 2010; Hooton et al., 2011; Chan and Abedon, 2012; Chan et al., 2013; Clavijo et al., 2019; Petsong et al., 2019). However, since levels of antimicrobial resistance were generally high in *Salmonella* spp. isolated from broilers, it is therefore necessary to investigate the effectiveness of phages and a developed phage cocktail against MDR *Salmonella* strains derived from broiler production chain, including broiler farms, slaughterhouses, and retail stores at the wet market.

Therefore, this study aims to evaluate the effective control strategy for *Salmonella* that were detected in broiler farms using phage cocktail. Environmental samples and cloacal swabs were screened for the occurrence of *Salmonella* in broiler farms from eastern and southern Thailand. Information from this study on the lysis ability of phages against *Salmonella* derived from broiler farms, MDR-*Salmonella* strains, and those major serovars linked to foodborne outbreaks will be useful for further phage selection and consideration of using phages as a potential biocontrol to improve food safety along broiler production or in poultry meat processing.

## Materials and Methods

### Broiler Farms and Sampling Location

Five broiler farms located in the eastern (*n* = 2) and southern (*n* = 3) areas of Thailand were included in this study. Broiler farms in this study are large flocks with over 20,000 birds. All farms are enclosed, located far away from human communities, natural reservoirs, and main roads, in a wide-open space. All farms have generally high infrastructure, high sanitary and hygienic conditions, and adequate biosecurity measures in controlling stray animals, rodents, reptiles, and amphibians. Waste and by-products are usually removed from a farm without contaminating the environment. In some farms, bedding materials are dried up and packaged within broiler houses before sold as manure.

### Collection of Samples

For two farms located in the east of Thailand, bedding materials composed of dried husk were collected by pooling sample (100 g) from four sections of a given pen on days 0, 2, 4, 6, 8, 9, 10, 15, 16, 20, 22, 23, 27, 31, 37, 39, and 42 of poultry cultivation. A total of 26 bedding samples were collected from farm A and farm B (Table 1). For three farms located in the south of Thailand, seven bedding samples were collected from farm C (*n* = 2), farm D (*n* = 2), and farm E (*n* = 3). Each sample was collected by pooling sample (100 g) from four sections of a given pen. Cloacal swabs were collected from farm C (*n* = 5), farm D (*n* = 6), and farm E (*n* = 5). Each cloacal sample was collected by swabbing 10 chickens with individual cotton stick, pooled as one sample, and transferred to a sterile zip-lock bag. Boot cover swab samples were collected from farm C (*n* = 5), farm D (*n* = 6), and farm E (*n* = 5). Each boot cover swab sample was collected by wearing boot covers and walking up and down each of the four sections of a given pen. All samples were kept with sterile plastic bags and stored in an icebox (4°C) during transportation to the laboratory for analysis.

**TABLE 1:** Distribution of *Salmonella* spp. in broiler farms.

Farm	No. of positive samples/No. of collected samples^*a*^ (%)			
	Bedding	Cloacal swab	Boot swab	Total
Eastern Farms				
Farm A	5/5	nc	nc	5/5 (100)
Farm B	7/21	nc	nc	7/21 (33.3)
Total	12/26 (46.2)	0	0	12/26 (46.2)
Southern Farms				
Farm C	2/3	3/5	2/2	7/10 (70)
Farm D	1/4	0/6	0/2	1/12 (8.3)
Farm E	1/2	0/5	0/3	1/10 (10)
Total	4/9 (44.4)	3/16 (18.8)	2/7 (28.6)	9/32 (28.1)

*^*a*^nc refers to no samples collected.*

### *Salmonella* Isolation and Confirmation

Collected samples were processed for *Salmonella* isolation following the protocol of Biomérieux company that was modified from ISO 6579:2002. Briefly, an approximately 25 g of bedding sample was enriched with 225 ml of buffered peptone water (BPW) (#421121, Biomérieux, Marcy l’Étoile, France) supplemented with Salmonella Supplement Tablet (#421202, Biomérieux, Marcy l’Étoile, France). For cloacal swabs and boot cover swabs, 90 ml of BPW supplemented with Salmonella Supplement Tablet was added to each sample. All samples were incubated at 41.5°C for 18 h. A loopful of each enriched sample was streaked on SALMA plate (#418247, Biomérieux, Marcy l’Étoile, France) and incubated at 37°C for 24 h. A pink to purple typical colony on the plate was observed and re-streaked on tryptic soy agar (TSA) plate to obtain a pure culture for further confirmation. *Salmonella* isolates were kept in 15% glycerol at -80°C for further analysis. Colonies of *Salmonella* were selected for serotyping by the agglutination latex test by a commercial service company (S. A. P. Laboratory Co., Ltd., Bangkok, Thailand).

### Bacteriophage Isolation, Purification, and Lysate Preparation

Wastewater samples from a wastewater treatment station of Prince of Songkla University hospital were collected for phage isolation. These include three samples of aerated wastewater treatment ponds (A1, A2, and A3) and two samples of sediment wastewater treatment pond (S1 and S2). Approximately 100 ml of each sample was kept in a sterile bottle and stored in an icebox (4°C) during transportation to the laboratory for analysis. One sample from a river (R) and another from a natural reservoir (NR) in Songkhla province were collected using sterile bottles and kept in an icebox during transportation to the laboratory. *Salmonella* phages were isolated using enrichment isolation with a multi-strain of *Salmonella* mixture obtained from broiler farms (Table 2) and from our laboratory collection (*S.* Agona H2-016 and *S.* Anatum A4-525). Enrichment and isolation steps were performed following a standard protocol from our laboratory (Petsong et al., 2019). Plaques were observed on each host lawn. A single plaque was chosen for purification for three passages with a specific host that showed a positive result, using a double-layer agar technique. Isolated plaque from the third purification passage was used to prepare 10-fold serial dilutions in SM buffer. Appropriate dilutions were used to prepare the overlay with the given host to yield the semi-confluent lysis and then harvest with 5 ml of SM buffer followed by centrifugation at 6000 rpm for 15 min at 4°C. The supernatant was filtrated through 0.20-μm syringe filters, and phage lysates were kept at 4°C. The phage titer was determined by counting plaques present on each plate of a given dilution (Petsong et al., 2019).

**TABLE 2:** Serovars of *Salmonella* derived from broiler farms.

Farm (no. of positive samples)	Type of sample	Serovar^*a*^	Code name
Eastern Farms			
Farm A (5)	Bedding	Schwarzengrund*^#^	H2
		Saintpaul*^#^	H13
		Albany*^#^	H32
		Kentucky*^#^	S1H28
		Kentucky	S2H28
Farm B (7)	Bedding	Mbandaka*^#^	H17D2
		Mbandaka	H1D8
		Mbandaka	H5D42
		Agona	H5D42
		Agona*^#^	H3D6
		Agona	H16D20
		Agona	H14D23
		Kentucky	H9D9
Southern Farms			
Farm C (7)	Bedding	Typhimurium	F1-W1-S1
		Typhimurium	F1-W1-S3
	Cloacal swab	Typhimurium^*#^	F1-W1-C2
		Typhimurium	F1-W1-C3
		Typhimurium	F1-W1-C4
	Boot swab	Typhimurium	F1-W1-B1
		Typhimurium	F1-W1-B2
Farm D (1)	Bedding	Agona^#^	F2-W3-S3
Farm E (1)	Bedding	Corvalis^*#^	F3-W5-S2

^*a*^Serovars with (*) indicate serovars used for phage enrichment and isolation. Serovars with (^#^) indicate serovars used for host range determination.

### Host Range Determination

Lysis ability for each phage was determined by a spot test on the bacterial lawn of a given *Salmonella* strain from broiler farms in this study, foodborne outbreak-linked *Salmonella* (*S.* Enteritidis S5-371, *S.* Hadar PPI-013, *S.* Infantis S5-506, *S.* Typhimurium S5-370, and *S.* Virchow H2-117), and the MDR-*Salmonella* strains listed in Supplementary Table 1. All selected MDR strains were previously isolated from several types of samples collected from the broiler production chain such as chicken meat stalls located in wet markets, slaughterhouses, commercial farms, and free range farms. All MDR isolates and their antibiotic resistance profiles were consequently investigated using a standard agar disk diffusion assay according to the Clinical and Laboratory Standard Institute guidelines (Clinical and Laboratory Standard Institute (CLSI), 2015; Sripaurya et al., 2019). Collection of *Salmonella* strains was kept at the Faculty of Agro-Industry, Prince of Songkla University. Lysis ability of isolated phages was determined by spotting 10 μl of each phage lysate (8 log PFU/ml) on the bacterial host lawn. Two independent replicates were performed. Phage lysis patterns were determined after 18–24 h of incubation at 37°C (Petsong et al., 2019).

### Efficiency of Plating

Three phage isolates with the highest % of lytic ability (see in Table 3) were selected for the assessment of efficiency of plating (EOP), following the protocol of Vongkamjan et al. (2017). Three phages were tested three times independently using four dilutions (3–6 log PFU/ml) against 24 different *Salmonella* isolates (eight serovars derived from broiler farms, five foodborne outbreak-related serovars, and 10 MDR serovars) and one of original strain for phage isolation. The EOP was calculated using the given formula:

**TABLE 3:** Lysis profiles of isolated *Salmonella* phages from various sources.

Descriptions	*Salmonella* phages (WP)									
	
	64	65	66	70	73	74	75.1	75.2	79.1	79.2
Host of isolation (refer to Table 2)	H2	H2	H2	H2	H13	H13	H13	H13	H13	H13
Source of isolation	A1	A2	S1	R	A1	A2	S1	S1	R	R
Plaque morphotype (mm)	0.5	0.5	0.5	0.5	0.5	0.5	1.0	0.5	1.0	0.5
Lysis profile (%)										
Eastern broiler farm isolates	66.7	66.7	66.7	66.7	50.0	50.0	50.0	50.0	50.0	50.0
Agona H3D6	+	+	+	+	−	−	−	−	−	−
Albany H32	−	−	−	−	−	−	−	−	−	−
Kentucky S1H28	+	+	+	+	+	+	+	+	+	+
Mbandaka H17D2	−	−	−	−	−	−	−	−	−	−
Saintpaul H13	+	+	+	+	+	+	+	+	+	+
Schwarzengrund H2	+	+	+	+	+	+	+	+	+	+
Southern broiler farm isolates	66.7	66.7	66.7	66.7	66.7	66.7	66.7	66.7	66.7	66.7
Agona F2-W3-S3	−	−	−	−	−	−	−	−	−	−
Corvalis F3-W5-S2	+	+	+	+	+	+	+	+	+	+
Typhimurium F1-W1-C2	+	+	+	+	+	+	+	+	+	+
Foodborne outbreak-related isolates	60.0	60.0	60.0	60.0	60.0	60.0	60.0	60.0	60.0	60.0
Enteritidis S5-371	+	+	+	+	+	+	+	+	+	+
Hadar PPI-013	−	−	−	−	−	−	−	−	−	−
Infantis S5-506	−	−	−	−	−	−	−	−	−	−
Typhimurium S5-370	+	+	+	+	+	+	+	+	+	+
Virchow H2-117	+	+	+	+	+	+	+	+	+	+
MDR isolates	81.8	81.8	81.8	81.8	81.8	81.8	81.8	77.3	81.8	81.8
Agona 223SL	+	+	+	+	+	+	+	−	+	+
Albany 198SL	+	+	+	+	+	+	+	+	+	+
Corvalis 069SL	−	−	−	−	−	−	−	−	−	−
Give 188SL	+	+	+	+	+	+	+	+	+	+
Kentucky 180SL	+	+	+	+	+	+	+	+	+	+
Kentucky 210SL	−	−	−	−	−	−	−	−	−	−
Kentucky 222SL	+	+	+	+	+	+	+	+	+	+
Kentucky 245SL	+	+	+	+	+	+	+	+	+	+
Kentucky 256SL	+	+	+	+	+	+	+	+	+	+
Mbandaka 034SL	−	−	−	−	−	−	−	−	−	−
Typhimurium 032SL	+	+	+	+	+	+	+	+	+	+
Typhimurium 205SL	+	+	+	+	+	+	+	+	+	+
Typhimurium 206SL	−	−	−	−	−	−	−	−	−	−
Schwarzengrund 086SL	+	+	+	+	+	+	+	+	+	+
Schwarzengrund 248SL	+	+	+	+	+	+	+	+	+	+
Schwarzengrund 252SL	+	+	+	+	+	+	+	+	+	+
Schwarzengrund 253SL	+	+	+	+	+	+	+	+	+	+
Singapore 154SL	+	+	+	+	+	+	+	+	+	+
Singapore 170SL	+	+	+	+	+	+	+	+	+	+
Singapore 174SL	+	+	+	+	+	+	+	+	+	+
Weltevreden 001SL	+	+	+	+	+	+	+	+	+	+
Weltevreden 013SL	+	+	+	+	+	+	+	+	+	+
% Total lysis ability	75.0	75.0	75.0	75.0	72.2	72.2	72.2	69.4	72.2	72.2
Host of isolation (refer to Table 2)	S1H28	S1H28	S1H28	A4-525	A4-525	A4-525	A4-525	A4-525	A4-525	H2-016
Source of isolation	A1	A2	S1	A1	A2	S1	S2	A3	NR	A2
Plaque morphotype (mm)	0.5	0.1	0.1	1.0	1.0	1.0	0.3	0.3	0.1	2.0
Lysis profile (%)										
Eastern broiler farm isolates	83.3	83.3	50.0	50.0	50.0	50.0	50.0	50.0	50.0	66.7
Agona H3D6	+	+	−	−	−	−	−	−	−	−
Albany H32	+	+	−	−	−	−	−	−	−	−
Kentucky S1H28	+	+	+	+	+	+	+	+	+	+
Mbandaka H17D2	−	−	−	−	−	−	−	−	−	+
Saintpaul H13	+	+	+	+	+	+	+	+	+	+
Schwarzengrund H2	+	+	+	+	+	+	+	+	+	+
Southern broiler farm isolates	66.7	66.7	66.7	66.7	66.7	66.7	66.7	66.7	66.7	66.7
Agona F2-W3-S3	−	−	−	−	−	−	−	−	−	−
Corvalis F3-W5-S2	+	+	+	+	+	+	+	+	+	+
Typhimurium F1-W1-C2	+	+	+	+	+	+	+	+	+	+
Foodborne outbreak-related isolates	100.0	80.0	80.0	60.0	60.0	60.0	60.0	60.0	60.0	80.0
Enteritidis S5-371	+	+	+	+	+	+	+	+	+	+
Hadar PPI-013	+	+	+	−	−	−	−	−	−	−
Infantis S5-506	+	−	−	−	−	−	−	−	−	+
Typhimurium S5-370	+	+	+	+	+	+	+	+	+	+
Virchow H2-117	+	+	+	+	+	+	+	+	+	+
MDR isolates	90.9	95.5	81.8	81.8	81.8	81.8	77.3	81.8	81.8	81.8
Agona 223SL	+	+	+	+	+	+	−	+	+	+
Albany 198SL	+	+	+	+	+	+	+	+	+	+
Corvalis 069SL	−	+	−	−	−	−	−	−	−	−
Give 188SL	+	+	+	+	+	+	+	+	+	+
Kentucky 180SL	+	+	+	+	+	+	+	+	+	+
Kentucky 210SL	+	+	−	−	−	−	−	−	−	−
Kentucky 222SL	+	+	+	+	+	+	+	+	+	+
Kentucky 245SL	+	+	+	+	+	+	+	+	+	+
Kentucky 256SL	+	+	+	+	+	+	+	+	+	+
Mbandaka 034SL	−	−	−	−	−	−	−	−	−	−
Typhimurium 032SL	+	+	+	+	+	+	+	+	+	+
Typhimurium 205SL	+	+	+	+	+	+	+	+	+	+
Typhimurium 206SL	+	+	−	−	−	−	−	−	−	−
Schwarzengrund 086SL	+	+	+	+	+	+	+	+	+	+
Schwarzengrund 248SL	+	+	+	+	+	+	+	+	+	+
Schwarzengrund 252SL	+	+	+	+	+	+	+	+	+	+
Schwarzengrund 253SL	+	+	+	+	+	+	+	+	+	+
Singapore 154SL	+	+	+	+	+	+	+	+	+	+
Singapore 170SL	+	+	+	+	+	+	+	+	+	+
Singapore 174SL	+	+	+	+	+	+	+	+	+	+
Weltevreden 001SL	+	+	+	+	+	+	+	+	+	+
Weltevreden 013SL	+	+	+	+	+	+	+	+	+	+
% Total lysis ability	88.9	88.9	75.0	72.2	72.2	72.2	69.4	72.2	72.2	77.8

The *asterisk* (*) indicates phages that were used in the phage cocktail development. A1, A2, and A3: water samples collected from aerated wastewater treatment ponds collected on March 28, 2019, September 17, 2019, and October 26, 2019, respectively. S1 and S2: water samples collected from sediment wastewater treatment ponds collected on March 28, 2019 and September 17, 2019, respectively. NR: water sample from natural reservoir, R: water sample from river in Songkhla province. Lysis ability: + represents lysis and − represents no lysis.

EOP = average PFU on target bacteria/average PFU on host bacteria

Efficiency of plating was classified as “High production” when the ratio was 0.5 or more. An EOP of 0.1 or higher, but below 0.5, was considered as “Medium production” efficiency, and that between 0.001 and 0.1 was considered as “Low production” efficiency. An EOP of 0.001 or below and when any dilutions did not result in any plaque formation were classified as inefficient (Mirzaei and Nilsson, 2015; Manohar et al., 2019).

### Transmission Electron Microscopy Analysis

Selected phages were identified as the morphology by transmission electron microscopy (TEM) analysis. Grid samples were prepared using a given phage lysate (8 log PFU/ml) following a protocol of Vongkamjan et al. (2012). Uranyl acetate (1%) was used for negative staining. The imaging was acquired with TEM model JEM-2010 (JEOL Ltd., Tokyo, Japan) at 160 kV and an instrumental magnification of 100,000× at Scientific Equipment Center, Prince of Songkla University, Hat Yai, Thailand.

### Development and Efficacy Evaluation of a Phage Cocktail

Three different phages with the highest lytic activity were mixed together in equal proportions (a ratio of 1:1:1) to obtain a phage cocktail stock at a concentration of 8 log PFU/ml. Selected phages used for phage cocktail development were chosen based on the highest % total lysis ability along with their lysis profile patterns against the strains derived from broiler farms and foodborne outbreak-related and MDR isolates. A developed phage cocktail was evaluated for its effectiveness on five strains linked to foodborne outbreaks and eight other strains presenting major serovars derived from broiler farms in Thailand (marked with ^∗^ in Table 2). A 20-ml suspension of a given strain (2 log CFU/ml) was mixed with 20 ml of a phage cocktail (final concentration of 5 and 7 log PFU/ml) at the ratio of 1:1 by volume [multiplicity of infection (MOI) 1000 and 100,000] and incubated at 37°C in a shaking incubator at 220 rpm for 18 h (Petsong et al., 2019). The culture broth was sampled every 6-h intervals for 18 h. Samples were serially 10-fold diluted with phosphate buffer saline (PBS) to obtain the appropriate dilution. Each dilution (0.1 ml) was spread on TSA plates. All plates were incubated at 37°C for 24–48 h. The culture of each *Salmonella* without a phage cocktail was served as control. All the tests were run in triplicate (Petsong et al., 2019). The presence and absence of *Salmonella* in the treatments and control were also confirmed by modified ISO 6579: 2002 following the protocol of Biomérieux company as previously described and re-streaked on Xylose Lysine Decarboxylase agar. The amount of phage during this assay was only monitored in the treatment of *S.* Enteritidis and *S.* Typhimurium as representatives by sampling the culture broth every 6-h intervals for 18 h. The culture broth was centrifuged at 6500 rpm for 15 min at 4°C and filtrated through 0.20-μm syringe filters. A number of phage was determined by counting plaques present on each plate of a given dilution as previously described.

In addition, the effectiveness of single phage treatment was also evaluated using *S.* Enteritidis S5-371 and *S.* Typhimurium S5-370 to compare the result with those of cocktail. A 20-ml *Salmonella* suspension (initial 3 log CFU/ml) was mixed with 20 ml of each phage or phage cocktail at a final concentration of 5 log PFU/ml (MOI 100). A number of viable *Salmonella* were examined as previously described.

### Evaluation of Phage-Resistant Development in *Salmonella* After Treatment With a Phage Cocktail

The change of *Salmonella* resistance phenotype after treatment with a cocktail was investigated by a spot test. *S.* Enteritidis S5-371 and *S.* Typhimurium S5-370 were used as representatives for all strains of *Salmonella* included in this study. The culture of a given *Salmonella* strain (5 log CFU/ml) was mixed with a phage cocktail (final concentration of 7 log PFU/ml) and incubated at 37°C in a shaking incubator at 220 rpm for 18 h. A loopful of culture was streaked on the TSA plate to obtain a single colony. A single colony was grown in TSB overnight as previously described. Each overnight culture was used to prepare a double layer plate for a spot test with a phage cocktail upon serial dilutions. Overnight culture was subsequently used for phage cocktail treatment in the second and third passage following the same protocol of the first passage to evaluate the phage resistance following the protocols of Petsong et al. (2019).

### Statistical Analysis

Statistical analysis was performed using SPSS (Version 22.0) of Windows statistics software (SPSS Inc., Chicago, IL, United States). Data of *Salmonella* reduction during incubation period were subjected to analysis of variance followed by Tukey’s range test. A significant difference between the control and the phage cocktail treatments was calculated using the independent-samples *t* test. A difference was considered statistically significant at *p* < 0.05.

## Results

### Overall Prevalence and Serovar Diversity of *Salmonella* spp. in Broiler Farms

*Salmonella* spp. were detected in 46.2 and 28.1% of samples collected from two broiler farms in eastern Thailand and three broiler farms in southern Thailand, respectively (Table 1). Bedding showed a typical source for *Salmonella* spp. in all farms sampled. Although only one sample type was collected in eastern farms due to contamination issue, all three sample types were collected in southern farms with only farm C showing *Salmonella* positive in three sample types (70% of samples collected).

Eastern farms showed higher diversity of *Salmonella* serovars from bedding samples than those from southern farms (Table 2). *Salmonella* detected from five positive samples from farm A presented four serovars including Schwarzengrund, Saintpaul, Albany, and Kentucky. Seven positive samples from farm B showed three serovars including Mbandaka, Agona, and Kentucky. Serovar Typhimurium was the only serovar detected in bedding, cloacal swab, and boot swab samples from farm C. Two positive bedding samples from farm D and farm E showed two distinct serovars, Agona and Corvalis.

### Phage Lysis Ability

Overall, 20 phages recovered from the collected water samples showed various plaque morphotypes, ranging from as tiny as 0.1 mm to a large plaque size as 2.0 mm (Table 3). Most phages isolated here (18 phages) showed similar lysis ability covering over 15 serovars from various sources. These presented 69.4–88.9% lysis ability on 36 different *Salmonella* isolates. In addition, two phages (WP109 and WP110) showed considerably strong lysis ability covering over 14 serovars from various sources and representing 88.9% lysis ability on 36 different *Salmonella* isolates.

All 20 phages showed strong lysis on the isolates presenting five major serovars, including Kentucky, Saintpaul, and Schwarzengrund from eastern farms and Corvalis and Typhimurium from southern farms. A few phages could lyse isolates of serovars Agona, Albany, and Mbandaka derived from broiler farms in this study. Only six phages (30%) could lyse *S.* Agona H3D6 isolated from bedding of farm B whereas no phage could lyse *S.* Agona F2-W3-S3 isolated from bedding of farm D (southern farm). Two phages (WP109 and WP110) showed strong lysis on *S.* Albany H32 isolated from bedding of farm A. The only phage WP128 showed strong lysis on *S.* Mbandaka H17D2 isolated from bedding of farm B.

All 20 phages showed strong lysis on the three reference serovars that were foodborne outbreak-related (Enteritidis, Typhimurium, and Virchow). *S.* Hadar PPI-013 was susceptible to lysis by phage WP109, WP110, and WP111 whereas serovar Infantis S5-506 was susceptible to lysis by two phages WP109 and WP128. Most phages isolated here showed strong lysis against MDR strains (10 serovars) representing 77.3–95.5% lysis ability on 22 different *Salmonella* isolates. Among 10 serovars of the MDR strains tested, only serovar Mbandaka showed to be resistant to all phages. One tetracycline-resistant strain, *S.* Corvalis 069SL, was susceptible to lysis by only phage WP110, whereas serovars Kentucky (210SL) and Typhimurium (206SL) were susceptible to lysis by two phages WP109 and WP110.

### Efficiency of Plating

The EOP assay reveals that phage WP109 showed high EOP on 6 of 24 isolates of *Salmonella* tested, medium EOP on 5 isolates, low EOP on 8 isolates, and an inefficient EOP on 5 isolates. Phage WP110 showed a high EOP on 6 isolates, medium EOP on 6 isolates, low EOP on 7 isolates, and an inefficient EOP on 4 isolates. Phage WP128 only showed high EOP against *S.* Agona H2-016, the original host and *S.* Agona 223SL. Interestingly, this phage could not produce a medium productive infection despite showing a high percentage of low EOP (10/24) and inefficient EOP (12/24) as shown in Table 4.

**TABLE 4:** Efficiency of plating (EOP) of selected phages on bacterial hosts.

*Salmonella*	Efficiency of plating (EOP)*		
	WP109	WP110	WP128
Agona H3D6	<0.001	<0.001	<0.001
Albany H32	<0.001	0.20 ± 0.00	<0.001
Kentucky S1H28	**1.00**	**1.00**	0.002 ± 0.00
Mbandaka H17D2	<0.001	<0.001	<0.001
Saintpaul H13	3.48 ± 0.74	1.00 ± 0.57	0.01 ± 0.00
Schwarzengrund H2	1.92 ± 0.11	0.43 ± 0.24	0.01 ± 0.00
Typhimurium F1-W1-C2	0.004 ± 0.00	0.002 ± 0.00	0.01 ± 0.00
Corvalis F3-W5-S2	0.004 ± 0.00	0.01 ± 0.00	<0.001
Enteritidis S5-371	8.90 ± 2.40	5.20 ± 1.13	0.02 ± 0.00
Hadar PPI-013	0.02 ± 0.00	0.008 ± 0.00	<0.001
Infantis S5-506	0.004 ± 0.00	<0.001	<0.001
Typhimurium S5-370	0.10 ± 0.03	0.01 ± 0.00	<0.001
Virchow H2-117	0.004 ± 0.00	0.02 ± 0.00	<0.001
Agona 223SL	0.004 ± 0.00	0.18 ± 0.00	1.31 ± 0.25
Albany 198SL	1.80 ± 0.28	1.42 ± 0.25	0.01 ± 0.00
Corvalis 069SL	<0.001	0.002 ± 0.00	<0.001
Give 188SL	0.45 ± 0.26	0.15 ± 0.07	<0.001
Kentucky 180SL	0.45 ± 0.26	2.90 ± 0.56	0.004 ± 0.00
Mbandaka 034SL	<0.001	<0.001	<0.001
Typhimurium 032SL	0.04 ± 0.01	0.07 ± 0.01	0.011 ± 0.001
Schwarzengrund 086SL	5.20 ± 0.00	0.40 ± 0.07	0.009 ± 0.00
Singapore 154SL	0.004 ± 0.00	0.002 ± 0.00	<0.001
Weltevreden 001SL	0.48 ± 0.08	0.14 ± 0.08	0.003 ± 0.00
Agona H2-016	0.26 ± 0.04	1.90 ± 0.14	**1.00**

*All values provided are expressed as mean ± standard deviation in triplicate. EOP values were classified as high production (EOP > 0.5), medium production (0.1 < EOP < 0.5), low production (0.001 < EOP < 0.1), and inefficient production (EOP < 0.001). The original strain of isolation has an EOP value of 1.0 and is shown in boldface (*S.* Kentucky H1S28 for WP109 and WP110, and *S.* Agona H2-016 for WP128).

### TEM Analysis

**FIGURE 1:**
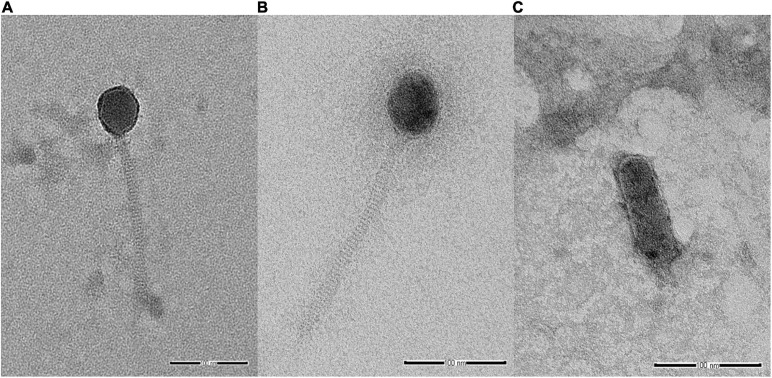
Transmission electron microscopy analysis of *Salmonella* phages. **(A)** vB_SenS_WP109, **(B)** vB_SenS_WP110, and **(C)** vB_SenP_WP128 at a magnification of 100,000×. Bar, 100 nm.

Analysis of selected *Salmonella* phages using TEM allowed the morphological classification of phages into viral order and family. *Salmonella* phages WP109 and WP110 were classified into the order *Caudovirales*, and family *Siphoviridae*. Phage WP109 showed icosahedral-shaped head with a size of 54.2 nm × 53.1 nm and a tail length of 182.1 nm (Figure 1A). Phage WP110 showed a head size of 59.8 nm × 55.3 mm and a tail length of 214.2 nm (Figure 1B). Phage WP128 was assigned to order *Caudovirales* and family *Podoviridae* with the C3 morphotype as shown in Figure 1C. This phage had an elongated head with a size of 96.9 nm × 32.2 nm and a short tail length of 19.2 nm.

### Evaluation of Phage Cocktail Efficiency Targeting *Salmonella in vitro*

To evaluate the efficiency of a phage cocktail on *Salmonella* growth, the number of *Salmonella* in the presence and absence of a phage cocktail was counted every 6-h interval for 18 h. As shown in Table 5, a phage-treated treatment could significantly reduce the cell number by 100% (2 log units) for strains *S.* Enteritis S5-371, *S.* Virchow H2-117, *S.* Schwarzengrund H2, *S.* Saintpaul H13, *S.* Kentucky S1H28, *S.* Typhimurium F1-W1-C2, and *S.* Corvalis F3-W5-S2 during 6 to 18 h in a phage cocktail treatment at MOI of 1000. In the treatment of higher MOI at 100,000, significant reduction of the cell number (100% reduction; 2 log units) was also observed for strains *S.* Typhimurium S5-370 and *S.* Hadar PPI-013. However, a phage cocktail incompletely reduced the population of *S.* Albany H32, *S.* Mbandaka H17D2, and *S.* Agona H3D6 derived from eastern broiler farm, and *S.* Infantis S5-506, the foodborne-outbreak isolate as observed by a reduction of only 0.1–0.3 and 0.1–1.3 log units after 6 h of treatment at MOI 1000 and 100,000, respectively.

**TABLE 5:** Efficacy evaluation of a phage cocktail on diverse *Salmonella* serovars.

*Salmonella*	Time (h)	Bacterial count^*A*^ (log CFU/ml)			%Reduction at MOI 1000	%Reduction at MOI 100000
		Control	Cocktail at MOI 1000	Cocktail at MOI 100000		
Agona H3D6	0	2.8 ± 0.2^*a*^	2.6 ± 0.1^*a*^	2.8 ± 0.2^*a*^	–	–
	6	6.1 ± 0.1^*b*^	5.8 ± 0.4^*b*^	5.7 ± 0.4^*b*^	16.3 ± 1.0	6.8 ± 0.5
	12	8.0 ± 0.0^*c*^	7.4 ± 0.1^*c*^	6.5 ± 0.2^c*^	7.4 ± 1.3	19.2 ± 2.7
	18	8.2 ± 0.2^*c*^	8.4 ± 0.2^*c*^	8.0 ± 0.1^*d*^	0	1.8 ± 0.1
Albany H32	0	2.3 ± 0.1^*a*^	2.3 ± 0.4^*a*^	2.7 ± 0.3^*a*^	–	–
	6	6.9 ± 0.1^*b*^	5.9 ± 0.1^b*^	6.1 ± 0.1^b*^	14.6 ± 1.3	11.4 ± 2.3
	12	8.1 ± 0.1^*c*^	7.3 ± 0.0^b*^	8.0 ± 0.0^*c*^	10.2 ± 0.9	2.0 ± 0.7
	18	8.4 ± 0.0^*d*^	8.2 ± 0.1^*c*^	8.0 ± 0.1^c*^	2.9 ± 1.3	5.5 ± 1.5
Kentucky S1H28	0	2.4 ± 0.6^*a*^	2.3 ± 0.2	2.2 ± 0.2	–	–
	6	7.2 ± 0.3^*b*^	nd	nd	100	100
	12	8.3 ± 0.1^*c*^	nd	nd	100	100
	18	8.1 ± 0.0^*c*^	nd	nd	100	100
Mbandaka H17D2	0	2.4 ± 0.1^*a*^	2.6 ± 0.2^*a*^	2.5 ± 0.2^*a*^	–	–
	6	6.7 ± 0.1^*b*^	6.6 ± 0.1^*b*^	6.6 ± 0.2^*b*^	1.4 ± 0.2	2.4 ± 0.3
	12	8.3 ± 0.0^*c*^	8.4 ± 0.3^*c*^	8.3 ± 0.1^*c*^	0	0
	18	8.1 ± 0.1^*c*^	9.5 ± 0.0^*d*^	8.2 ± 0.0^*c*^	0	0
Saintpaul H13	0	2.1 ± 0.1^*a*^	2.2 ± 0.2	2.4 ± 0.4	–	–
	6	6.5 ± 0.2^*b*^	nd	nd	100	100
	12	8.4 ± 0.1^*c*^	nd	nd	100	100
	18	8.4 ± 0.1^*c*^	nd	nd	100	100
Schwarzengrund H2	0	2.3 ± 0.3^*a*^	2.4 ± 0.3	2.4 ± 0.4	–	–
	6	6.4 ± 0.1^*b*^	nd	nd	100	100
	12	8.3 ± 0.1^*c*^	nd	nd	100	100
	18	8.3 ± 0.1^*c*^	nd	nd	100	100
Typhimurium F1-W1-C2	0	2.9 ± 0.5^*a*^	2.3 ± 0.1	2.6 ± 0.1	–	–
	6	6.4 ± 0.3^*b*^	nd	nd	100	100
	12	8.2 ± 0.0^*c*^	nd	nd	100	100
	18	8.3 ± 0.0^*d*^	nd	nd	100	100
Corvalis F3-W5-S2	0	2.3 ± 0.0^*a*^	2.3 ± 0.2	2.3 ± 0.0	–	–
	6	6.7 ± 0.2^*b*^	nd	nd	100	100
	12	8.3 ± 0.1^*c*^	nd	nd	100	100
	18	8.5 ± 0.2^*d*^	nd	nd	100	100
Enteritidis S5-371	0	2.4 ± 0.3^*a*^	2.4 ± 0.2	2.4 ± 0.3	–	–
	6	6.9 ± 0.1^*b*^	nd	nd	100	100
	12	8.3 ± 0.1^*c*^	nd	nd	100	100
	18	8.3 ± 0.1^*c*^	nd	nd	100	100
Hadar PPI-013	0	2.9 ± 0.2^*a*^	2.9 ± 0.2^*a*^	2.7 ± 0.3	–	–
	6	6.5 ± 0.8^*b*^	3.4 ± 0.2^a*^	nd	47.1 ± 5.0	100
	12	8.2 ± 0.1^*c*^	3.6 ± 0.1^a*^	nd	55.9 ± 1.8	100
	18	8.5 ± 0.2^*c*^	9.1 ± 0.1^*b*^	nd	0	100
Infantis S5-506	0	2.4 ± 0.2^*a*^	2.6 ± 0.1^*a*^	2.2 ± 0.0^*a*^	–	–
	6	6.8 ± 0.2^*b*^	6.7 ± 0.2^*b*^	5.5 ± 0.1^b*^	0.6 ± 0.1	18.4 ± 3.0
	12	8.2 ± 0.0^*c*^	7.5 ± 0.4^*b*^	6.8 ± 0.1^c*^	9.1 ± 0.9	16.6 ± 0.9
	18	8.3 ± 0.3^*c*^	9.3 ± 0.1^*c*^	8.0 ± 0.1^*d*^	0	3.3 ± 0.8
Typhimurium S5-370	0	2.3 ± 0.3^*a*^	2.4 ± 0.3^*a*^	2.3 ± 0.1	–	–
	6	6.4 ± 0.1^*b*^	2.3 ± 0.2^a*^	nd	66.0 ± 2.2	100
	12	8.3 ± 0.1^*c*^	4.6 ± 0.0^b*^	nd	45.0 ± 0.3	100
	18	8.3 ± 0.1^*c*^	7.3 ± 0.0^c*^	nd	12 ± 0.1	100
Virchow H2-117	0	2.6 ± 0.1^*a*^	2.7 ± 0.1	2.7 ± 0.0	–	–
	6	6.9 ± 0.0^*b*^	nd	nd	100	100
	12	8.3 ± 0.0^*c*^	nd	nd	100	100
	18	8.4 ± 0.1^*c*^	nd	nd	100	100

^*A*^All values provided are expressed as mean ± standard deviation in triplicate. The lowercase letters for control or phage treatment and those connected by different letters are significantly different (*p* < 0.05) whereas the *asterisk* (*) indicates the significant difference (*p* < 0.05) of bacterial counts between control and the phage treatment at the same time. “nd” refers to no bacterial count detected. The percentage of bacterial reduction at 0 h was normalized to 0.0.

**FIGURE 2:**
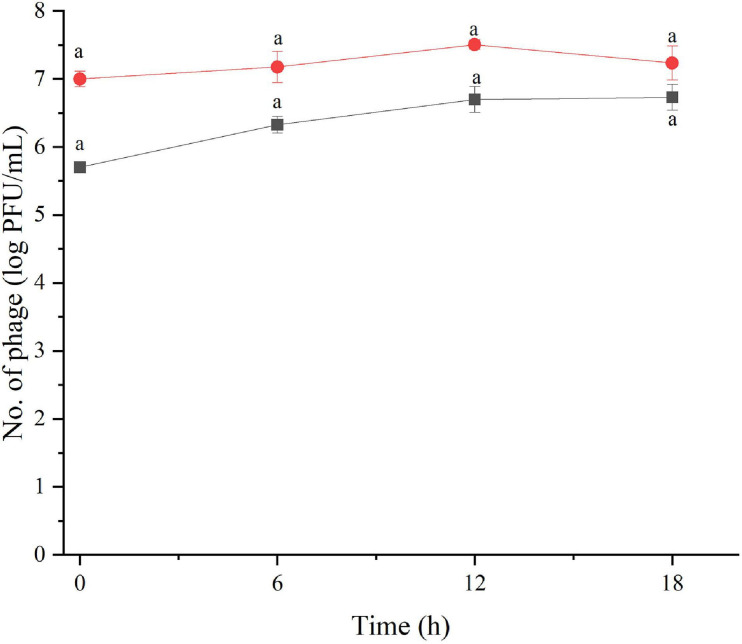
The number of phage concentration during efficacy evaluation of a phage cocktail on *Salmonella* Enteritidis. MOI100 (black square and line) and MOI 100000 (red circle and line). All values provided are expressed as mean ± standard deviation in triplicate. The lowercase letters connected by the different letters are significantly different (*p* < 0.05).

**FIGURE 3:**
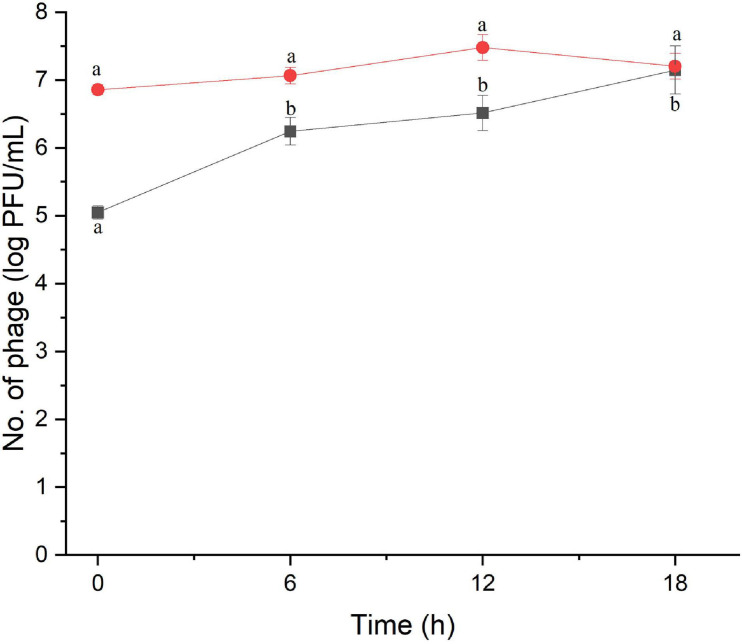
The number of phage concentration during efficacy evaluation of a phage cocktail on *Salmonella* Typhimurium. MOI 1000 (black square and line) and MOI 100000 (red circle and line). All values provided are expressed as mean ± standard deviation in triplicate. The lowercase letters connected by the different letters are significantly different (*p* < 0.05).

The amount of phage during the assay was also monitored as shown in Figures 2, 3. The results indicated that the number of phages slightly increased in the treatment of *S.* Enteritidis at both MOIs (5.6–6.7 PFU/ml at MOI 1000 and 7.0–7.5 at MOI 100,000) but without significant difference at each sampling time (*p* > 0.05) as shown in Figure 2. In the treatment of *S.* Typhimurium, the number of phages significantly increased at MOI 1000 (*p* < 0.05) (5.0–7.1 log PFU/ml) whereas no significant difference was observed at MOI 100,000 (6.9–7.2 log PFU/ml) (Figure 3).

**FIGURE 4:**
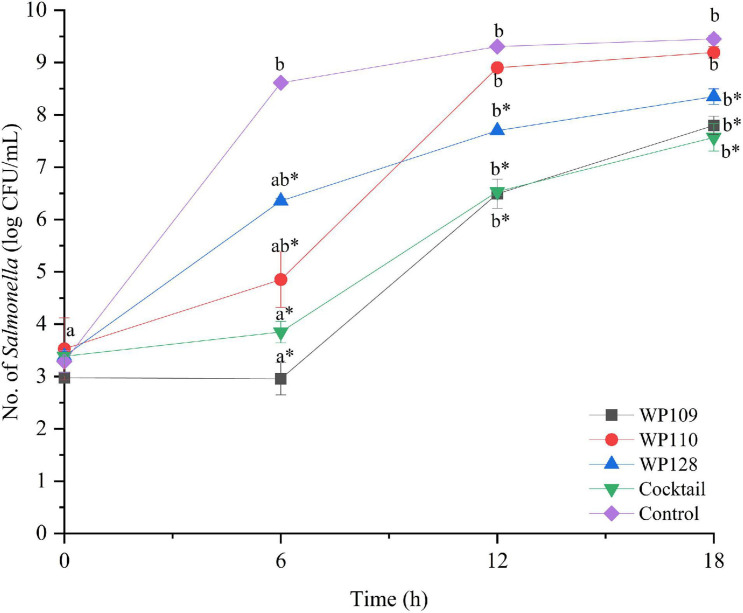
The number of viable *Salmonella* Enteritidis count when treated with a phage cocktail (5 log PFU/ml) with the beginning of bacterial concentration by 3 log CFU/ml (MOI 100). All values provided are expressed as mean ± standard deviation in triplicate. The lowercase letters for control or phage treatment and those connected by the different letters are significantly different (*p* < 0.05) whereas the *asterisk* (*) indicates the significant difference (*p* < 0.05) of bacterial counts between control and the phage treatment at the same time.

**FIGURE 5:**
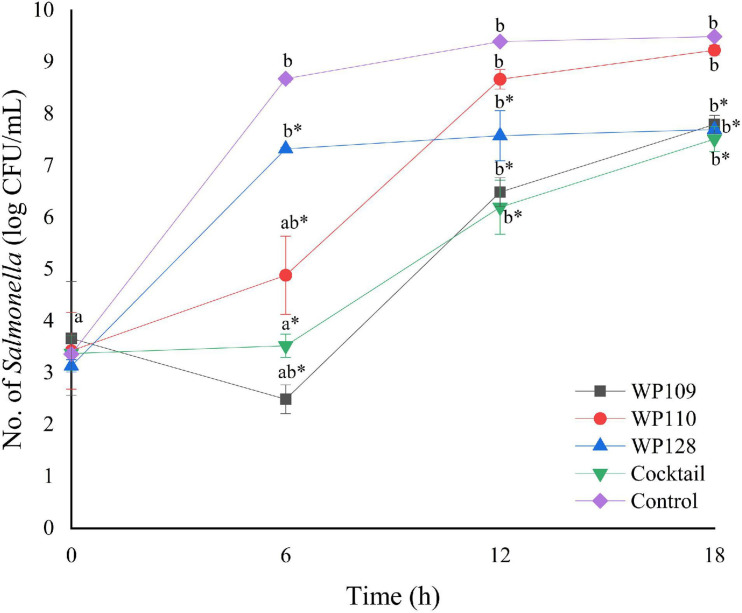
The number of viable *Salmonella* Typhimurium count when treated with a phage cocktail (5 log PFU/ml) with the beginning of bacterial concentration by 3 log CFU/ml (MOI 100). All values provided are expressed as mean ± standard deviation in triplicate. The lowercase letters for control or phage treatment and those connected by the different letters are significantly different (*p* < 0.05) whereas the *asterisk* (*) indicates the significant difference (*p* < 0.05) of bacterial counts between control and the phage treatment at the same time.

The study of bacterial inactivation by each of the three single phages was performed. The significant difference was observed in each single phage and a phage cocktail at 6 h (*p* < 0.05) for both *Salmonella*. The significant reduction of *Salmonella* cells was observed in the treatment of phage WP109 or phage WP128, and phage cocktail at 12 and 18 h (*p* < 0.05) when compared to the control. However, phage WP110 could reduce the number of both *Salmonella* at 12 and 18 h but there was no significant difference (*p* > 0.05). Importantly, phage cocktail showed higher reduction of *Salmonella* counts than other single phage treatments at the end of study as shown in Figures 4, 5.

### Evaluation of Phage-Resistance Development in *Salmonella* After Treatment With a Phage Cocktail

After treatment with a phage cocktail, *S.* Enteritis and *S.* Typhimurium culture were re-tested with the same phage cocktail for three passages. Compared to the control culture, which had no prior phage cocktail treatment, the phage-treated *Salmonella* culture showed the same lysis ability with a phage cocktail for both serovars and for three passages (Table 6).

**TABLE 6:** Re-challenge of *S.* Enteritidis and *S.* Typhimurium upon phage treatment by a phage cocktail.

Treatments	Lysis ability^*a*^ of cocktail (Phage titer log PFU/ml)											
	Passages											
	1				2				3			
	
	7	6	5	4	7	6	5	4	7	6	5	4
Control *S.* Enteritidis	+++	++	++	+	++	++	+	−	++	++	+	−
Cocktail-treated *S.* Enteritidis	+++	++	++	++	++	++	+	−	++	++	+	−
Control *S.* Typhimurium	++	+	+	−	+++	++	+	−	+++	++	+	−
Cocktail-treated *S.* Typhimurium	+++	++	+	+	++	++	+	−	+++	++	+	−

*^*a*^Lysis ability: +++ indicates strong lysis; ++ indicates medium lysis; + indicates weak lysis; − indicates no lysis. Control is culture without prior phage cocktail treatment.*

## Discussion

Several studies have been reported showing that samples such as bedding materials, feed, water, cloacal swab, boot swab, dust, and even litter collected from several regions of commercial poultry farms worldwide are important sources of *Salmonella* spp. (Mueller-Doblies et al., 2009; Djeffal et al., 2018; Eguale, 2018; Dagnew et al., 2020). Various contamination rates with *Salmonella* were observed, for example, in European countries; 68 of 4331 samples (1.57%) were collected from poultry farms in the region of Pomerania, Warmia, and Mazury of Northern Poland during 2014 to 2016 (Witkowska et al., 2018). In African countries, 370 samples (14.1%) including dust, litter, feces, feed, and water samples collected from 228 poultry farms in different regions of Nigeria showed a positive result for *Salmonella* isolation (Fagbamila et al., 2017), and 4.7% of collected samples (pooled fresh fecal dropping) from 48 examined poultry farms in Ethiopia from July 2013 to January 2014 were also positive for *Salmonella* (Eguale, 2018).

The colonization of *Salmonella* at the farm level has been considered as the most high-risk factor for the human food chain. It is linked with the contamination of poultry products and causes the possible outbreak of human salmonellosis. In the present study, 21 of 58 samples (36.20%) from five examined poultry farms were contaminated by *Salmonella*. The prevalence of *Salmonella* spp. observed here was higher than the above reports (Ziyate et al., 2016; Fagbamila et al., 2017; Eguale, 2018). The possible reason could be due to differences in farm management and practices as all examined farms were large-scale commercial poultry farms that hold more than 20,000 chickens per cultivation. Furthermore, the accumulation of this pathogen in bedding materials by excreting this pathogen through the feces of chickens without any changing materials is also an important factor (Nair and Johny, 2019), thus showing as *Salmonella*-positive in bedding samples from all examined farms.

Many serovars of *Salmonella*, especially the non-typhoidal *Salmonella*, have been found among poultry farms in several parts of the world, which can lead to contamination in poultry products such as fresh meat and raw eggs (Kumar et al., 2019). For example, the dominant serovar of *Salmonella* distributed in poultry farms in Ethiopia was *S.* Saintpaul (76.92%), followed by *S.* Typhimurium (11.53%), *S.* Kentucky (7.69%), and *S.* Haifa (3.85%) (Eguale, 2018). *S.* Kentucky was frequently isolated from poultry farms in Nigeria and Bangladesh (Barua et al., 2012; Fagbamila et al., 2017), while seven different serovars were distributed in laying hen farms in Morocco as follows: *S.* Enteritidis (37.5%), *S.* Kentucky (31.3%), *S.* Infantis (10.9%), *S.* Typhimurium (6.24%), *S.* Thompson (6.2%), *S.* Agona (4.7%), and *S.* Amsterdam (3.1%) (Ziyate et al., 2016). In Thailand, *S.* Corvalis (37.8%), *S.* Albany (24.3%), and *S.* Enteritidis (24.3%) are the most serovars isolated from broiler farms in Chiang Mai province (Lampang et al., 2014). *S.* Kentucky (22.94%) and *S.* Give (20.18%) were also found as the most serovars in local slaughterhouses in nine provinces of central Thailand between April and July 2018 (Phongaran et al., 2019). Similar to the previous reports, *S.* Agona, *S.* Kentucky, *S.* Mbandaka, and *S.* Typhimurium observed in this study were the major serovars among samples collected from poultry farms, while *S.* Albany, *S.* Schwarzengrund, *S.* Corvalis, and *S.* Saintpaul presented as minor serovars.

A number of *Salmonella* phages could be isolated from various water sources in this study, suggesting that water and/or wastewater are common sources of *Salmonella* phages as previously reported by several studies (Sundar et al., 2009; Rattanachaikunsopon and Phumkhachorn, 2012; Yildirim et al., 2018; Petsong et al., 2019). Previous studies have shown the usefulness of phage therapy using phages with a board-lysis ability, including those five *Salmonella* phages (phiSE) isolated from chicken feces that could lyse six important serovars tested including Abony, Enteritidis, Gallinarum, Pullorum, Typhi, and Typhimurium (Hungaro et al., 2013). *Salmonella* phages STm101 and STm118 isolated from Thai poultry farms could also infect eight different *Salmonella* serovars (infected 50% of tested isolates) (Phothaworn et al., 2019). In addition, MDR isolates have been critical for animal farms. Previous studies have focused on using phages to target MDR isolates such as seven virulent *Salmonella* phages (SPFM) that could infect 100% of MDR-*Salmonella* isolated from pigs in the United Kingdom (Thanki et al., 2019) whereas other six *Salmonella* phages could lyse clinically isolated and ciprofloxacin-induced antibiotic-resistant *S.* Typhimurium (Jung et al., 2017). In the current study, 20 isolated phages showed strong lysis patterns against serovars from broiler farms, serovars with food-linked illness, and MDR strains, suggesting that their lytic activities are advantageous in combating *Salmonella* prevalent in the broiler production chain and preventing MDR spread through the food chain.

Phages with a board-host lysis ability are able to eliminate certain hosts due to their high specificity. The EOP assay confirmed that individual phages included in a phage cocktail showed high productive infection against most isolates of *Salmonella* from diverse sources in this study. The EOP assay has been widely used for phage efficacy evaluation (Mirzaei and Nilsson, 2015; Manohar et al., 2019). In this study, single phage treatments could reduce *Salmonella* counts. However, the phage cocktail showed higher reduction of *Salmonella* counts than other single phage treatments. The combination of several phages in treatment as a phage cocktail is usually preferred and used for treating the bacterial co-infections and can expand their lytic activity as previous reported by others (Goodridge, 2010; Hooton et al., 2011; Chan and Abedon, 2012; Rattanachaikunsopon and Phumkhachorn, 2012; Chan et al., 2013). In addition, a single phage can increase the chance of phage resistance in bacteria compared to phage cocktail treatment. This is because the bacteria might resist one type of phage in cocktail but is still susceptible to others as previously reported by others (Rohde et al., 2018; Yang et al., 2020). In a previous work, the *in vitro* study showed that a phage cocktail made of KP4, KP5, and KP50 could decrease the number of *S.* Enteritidis and *S.* Typhimurium by 4 log CFU/ml after 4 h of treatment (Petsong et al., 2019). SalmoLyse, the cocktail of six *Salmonella* phages, at concentration levels of 8 log PFU/ml or greater, was able to inactivate 90% of *S.* Typhimurium growth after 1 h of incubation (Heyse et al., 2015). In the current study, our newly developed phage cocktail could reduce the number of tested *Salmonella* serovars derived from foodborne outbreak-related and animal farm origin as indicated by the highest reduction by 100% after 6 h of treatment with MOI 1000 and 100,000. On the other hand, some isolates including *S.* Albany H32, *S.* Mbandaka H17D2, *S.* Agona H3D6, and *S.* Infantis S5-506 seem to be resilient to phage cocktail treatment since these isolates were susceptible to only one or two phages composed in this cocktail despite the fact that each isolate was treated with low MOI. However, phage cocktail could reduce the number of these isolate counts, but there was no significant difference (*p* > 0.05). The amount of phages during phage cocktail efficiency assay might correlate with the number of viable *Salmonella* count (Table 5). In the treatment of *S.* Enteritidis, both MOIs could reduce the number of *Salmonella* by 100% reduction at 6 h. This is why the number of phage was not rapidly produced. In contrast to the treatment of *S.* Enteritidis at MOI 1000, this MOI could not completely reduce the number of *Salmonella* during assay. This is why the number of phages was significantly increased. At MOI 100,000, the number of phages remained in the concentration between 6.9 and 7.2 log PFU/ml, and this result is concordant to the 100% reduction of bacteria at 6 h.

In addition, phage cocktail did not change *Salmonella* phenotypic characteristics through resistant patterns after treatment with this phage cocktail, indicating that no mutation was observed. Therefore, the use of this phage cocktail could be specific for controlling *Salmonella* distributed/contaminated in the broiler production chain and MDR *Salmonella*.

## Conclusion

Multiple *Salmonella* serovars have been prevalent in broiler farms from eastern and southern Thailand. The collected data could be used as an effective tool to explore the alternative strategy to combat *Salmonella* contamination and/or infection in broiler production chain. In addition, the overuse of antibiotics during broiler production can impact the development of resistance in bacteria and subsequently pose a serious threat to humans through the food production chain. Phages should be an attractive strategy as indicated by their specific lysis ability on the *Salmonella* hosts. Hence, *Salmonella* phages isolated in this study were tested against *Salmonella* hosts that may cause potential food safety issues in the broiler production chain. Overall, these phages showed strong lysis on the MDR strains, indicating potential phage control application to prevent the spread of MDR strains in the broiler production chain. Our study showed that a newly developed phage cocktail can decrease and/or eliminate *Salmonella* spp. *in vitro*. However, the efficiency of this cocktail application on a large scale should be undertaken in the further work. The real impact of utilization of phage cocktail as a biocontrol agent on poultry meat production and for preventing the spread of MDR through food production chain needs to be evaluated.

## Data Availability Statement

The raw data supporting the conclusions of this article will be made available by the authors, without undue reservation.

## Author Contributions

All authors conceived and designed the experiments and approved the final draft of the manuscript. WP executed the lab experiments, analyzed the data, and prepared the manuscript. KV is the principal investigator of the project who was responsible for preparation of project proposal, procuring funding, resource allocation, and managing human resource along with RN, MY, KC, and SB.

## Conflict of Interest

The authors declare that the research was conducted in the absence of any commercial or financial relationships that could be construed as a potential conflict of interest.

## Supplementary Material

Supplementary Table 1 Antibiotic-resistant *Salmonella* used in host range determination.
