# Supplementary material for: Isolation and Characterization of Potential Salmonella Phages Targeting Multidrug-Resistant and Major Serovars of Salmonella Derived From Broiler Production Chain in Thailand
Source: Front Microbiol. 2021 May 28;12:662461. doi: 10.3389/fmicb.2021.662461 (PMC8195598; doi:10.3389/fmicb.2021.662461)
Supplement: Supplementary Table 1 — Antibiotic-resistant Salmonella used in host range determination. [file Table_1.DOCX]

Supplementary Material

**Table S1** Antibiotic-resistant *Salmonella* used in host range determination

| **MDR *Salmonella*** | **Code** | **Sources of isolation** | **Resistance profile ^a^** |
| --- | --- | --- | --- |
| Agona | 223SL | Thigh (chicken meat) | AMP, S, TE, SXT |
| Albany | 198SL | Food contact surface (working table) | AMP, TE, C, SXT |
| Corvalis | 069SL | Food contact surface (working table) | TE |
| Give | 188SL | Slaughterhouse wastewater | AMP, S, NA, SXT |
| Kentucky | 180SL | Slaughterhouse wastewater | AMP, TE, CIP, NA |
|  | 210SL | Food contact surface (scale) | AMP, CIP, NA |
|  | 222SL | Drumstick (chicken meat) | AMP, S, CN, TE, CIP, NA |
|  | 245SL | Feces collected from free-ranged farm | AMP, S, TE, CIP, NA, SXT |
|  | 256SL | Slaughterhouse | AMP, S, CN, TE, NA |
| Mbandaka | 034SL | Soil collected from farm | S, TE, SXT |
| Typhimurium | 032SL | Animal feed | AMP, S, TE |
|  | 205SL | Food contact surface (working table) | AMP, S, TE, C, SXT |
|  | 206SL | Food contact surface (working table) | AMP, S, TE, NA, SXT |
| Schwarzengrund | 086SL | Food contact surface (cutting board) | AMP, S, CN, NA, C, SXT |
|  | 248SL | Slaughterhouse bucket | AMP, S, CN, TE, NA, C, SXT |
|  | 252SL | Slaughterhouse grid | AMP, S, CN, TE, SXT |
|  | 253SL | Slaughter area | AMP, CN, TE, NA, C, SXT |
| Singapore | 154SL | Plucking machine | AMP, TE, NA |
|  | 170SL | Slaughterhouse conveyor | AMP, S, TE, NA, CRO |
|  | 174SL | Slaughterhouse conveyor | AMP, S, TE, NA |
| Weltevreden | 001SL | Cooling pad water | AMP, C |
|  | 013SL | Cooling pad water | AMP, S, TE, SXT |

^a^ List of antibiotic abbreviation: AMP, ampicillin; S, streptomycin; CN, cefotetan; TE, tetracycline; CIP, ciprofloxacin; NA, nalidixic acid; C, chloramphenicol; SXT, trimethoprim-sulphamethoxazole; CRO, ceftriaxone
